# SRC kinase activator CDCP1 promotes hepatocyte growth factor–induced cell migration/invasion of a subset of breast cancer cells

**DOI:** 10.1016/j.jbc.2022.101630

**Published:** 2022-01-24

**Authors:** Naoyuki Kawase, Atsuya Sugihara, Kentaro Kajiwara, Michio Hiroshima, Kanako Akamatsu, Shigeyuki Nada, Kunio Matsumoto, Masahiro Ueda, Masato Okada

**Affiliations:** 1 Department of Oncogene Research, Research Institute for Microbial Diseases, Osaka University, Suita, Osaka, Japan; 2 Laboratory for Cell Signaling Dynamics, RIKEN Center for Biosystems Dynamics Research, Suita, Osaka, Japan; 3 Laboratory of Oncogene Research, World Premier International Immunology Frontier Research Centre, Osaka University, Suita, Osaka, Japan; 4 Division of Tumor Dynamics and Regulation, Cancer Research Institute, Kanazawa University, Kanazawa, Japan; 5 Laboratory of Single Molecule Biology, Graduate School of Frontier Biosciences, Osaka University, Suita, Osaka, Japan; 6 Center for Infectious Disease Education and Research, Osaka University, Suita, Osaka, Japan

**Keywords:** HGF, MET, CDCP1, ARHGEF7, RAC1, cell migration, invasion, cancer cell, CDCP1, CUB domain-containing protein 1, FBS, fetal bovine serum, GEF, guanine nucleotide exchange factor, HGF, hepatocyte growth factor, PFA, paraformaldehyde, PI3K, phosphatidylinositol 3-kinase, PIP3, phosphatidylinositol 3,4,5-trisphosphate, SFK, Src family kinase

## Abstract

Cancer invasion and metastasis are the major causes of cancer patient mortality. Various growth factors, including hepatocyte growth factor (HGF), are known to promote cancer invasion and metastasis, but the regulatory mechanisms involved are not fully understood. Here, we show that HGF-promoted migration and invasion of breast cancer cells are regulated by CUB domain–containing protein 1 (CDCP1), a transmembrane activator of SRC kinase. In metastatic human breast cancer cell line MDA-MB-231, which highly expresses the HGF receptor MET and CDCP1, we show that *CDCP1* knockdown attenuated HGF-induced MET activation, followed by suppression of lamellipodia formation and cell migration/invasion. In contrast, in the low invasive/nonmetastatic breast cancer cell line T47D, which had no detectable MET and CDCP1 expression, ectopic MET expression stimulated the HGF-dependent activation of invasive activity, and concomitant CDCP1 expression activated SRC and further promoted invasive activity. In these cells, CDCP1 expression dramatically activated HGF-induced membrane remodeling, which was accompanied by activation of the small GTPase Rac1. Analysis of guanine nucleotide exchange factors revealed that ARHGEF7 was specifically required for CDCP1-dependent induction of HGF-induced invasive ability. Furthermore, immunofluorescence staining demonstrated that CDCP1 coaccumulated with ARHGEF7. Finally, we confirmed that the CDCP1-SRC axis was also crucial for HGF and ARHGEF7-RAC1 signaling in MDA-MB-231 cells. Altogether, these results demonstrate that the CDCP1-SRC-ARHGEF7-RAC1 pathway plays an important role in the HGF-induced invasion of a subset of breast cancer cells.

Cancer invasion and metastasis are considered major causes of cancer-related mortality. As cancer progresses, malignant cancer cells break the basal membrane, invade the stroma, and enter the circulatory system to metastasize distantly. During these processes, various growth factors released from the tumor microenvironment contribute to promoting cancer cell invasion and metastasis. The hepatocyte growth factor (HGF) is a growth factor that promotes cancer malignancy. HGF was originally identified as a mitogenic factor for hepatocytes (1) and scatter factor (2, 3). In addition, MET has been identified as a receptor tyrosine kinase for HGF (4). HGF binding promotes MET dimerization to activate autophosphorylation, resulting in the recruitment of various adaptor proteins and signal transducers, such as STAT3, Akt, MAPK, and Src (5). Through activation of these multiple pathways, HGF–MET controls diverse physiological responses, including developmental morphogenesis, tissue regeneration, and organ homeostasis (6, 7). In particular, HGF plays a crucial role in dynamic cell migration and survival (8).

HGF and MET are aberrantly upregulated in various cancers, such as breast and esophageal cancers, hepatocellular carcinoma, and non–small cell lung cancer (9). Persistent activation of HGF-induced phenotypes contributes to the progression of invasion and metastasis, as well as the growth and survival of cancer cells. Thus, HGF–MET signaling has been considered a promising therapeutic target for a subset of cancers (10, 11). However, the mechanism of continuous activation of the HGF–MET pathway in invasive and metastatic cancer cells remains unclear.

In a previous study, we showed that CUB domain–containing protein 1 (CDCP1) is crucial for regulating HGF-MET signaling in normal epithelial Madin–Darby canine kidney cells (12). CDCP1 is a transmembrane scaffold of Src tyrosine kinase, and its upregulation has been implicated in tumor progression (13, 14, 15, 16), particularly cancer invasion and metastasis (15, 17, 18, 19). Our analysis using Madin–Darby canine kidney cells has revealed that CDCP1 functionally interacts with MET in membrane microdomains and promotes the activation of the MET-STAT3 pathway that transcriptionally induces invasive properties, such as the production of matrix metalloproteases (12). However, CDCP1’s requirement for HGF-induced invasion and metastasis of human cancer cells was undetermined. Furthermore, the contribution of pathways other than the MET-STAT3 axis needs to be investigated.

To address the above issues, we used the metastatic human breast cancer cell line MDA-MB-231, which has abundant MET and CDCP1 expression, and a low invasive/nonmetastatic breast cancer cell line T47D, which has no detectable expression of both mentioned proteins, as model systems. We demonstrated that CDCP1 promoted HGF-induced migration and invasion of breast cancer cells. A mechanistic analysis further revealed that CDCP1 activated the ARHGEF7-RAC1 axis to promote HGF-induced cytoskeleton and membrane remodeling, a prerequisite for cell migration and invasion. These findings shed light on the function of CDCP1 in regulating HGF-MET signaling in cancer invasion and metastasis.

## Results

### CDCP1 is required for HGF-promoted cell migration and invasion in human breast cancer cell line MDA-MB-231

**Figure 1:**
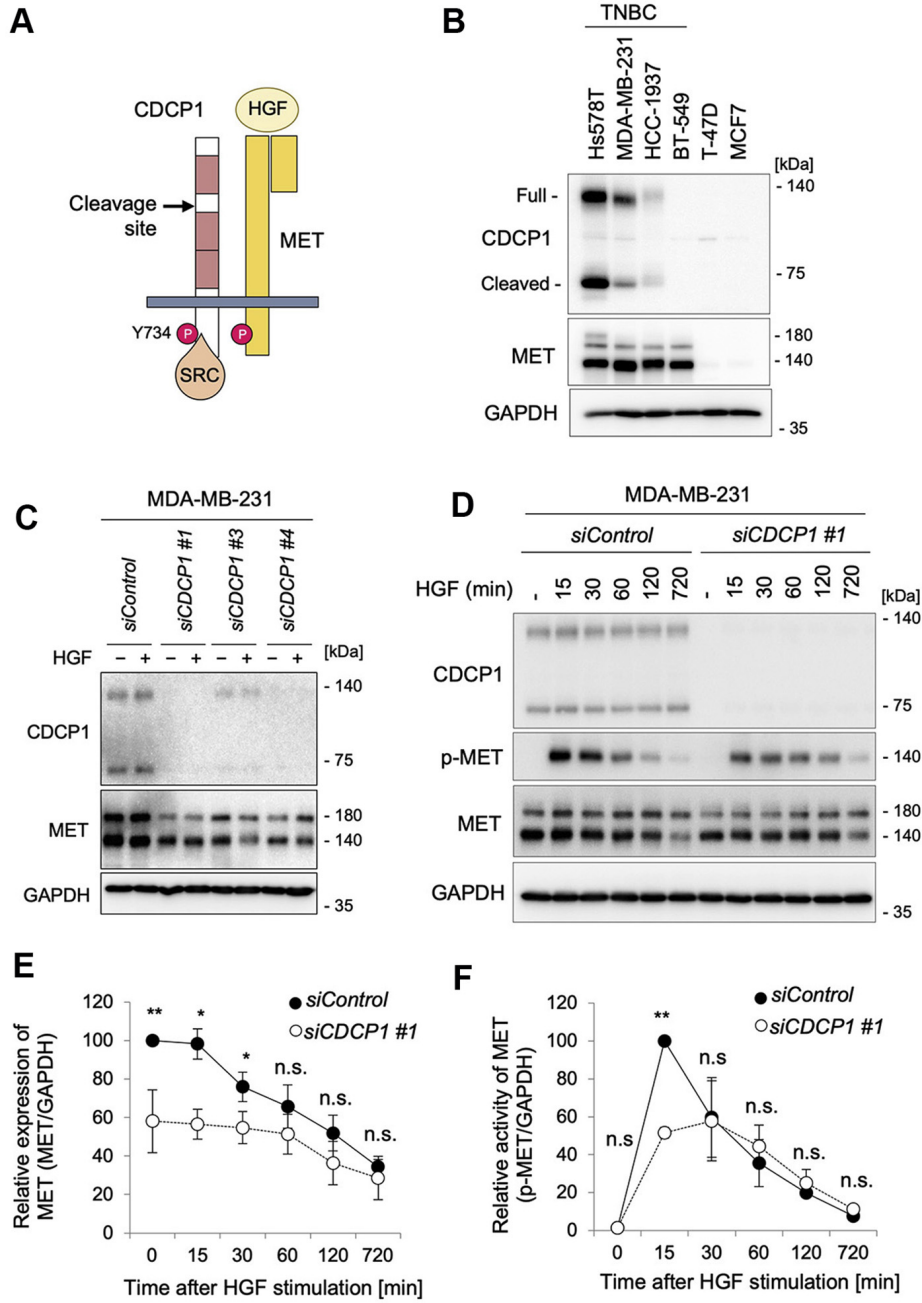
**CDCP1 functionally interacts with MET in human breast cancer cell line MDA-MB-231.***A*, schematic diagram of the structures of CDCP1 and MET. *B*, immunoblot analysis for the expression of CDCP1 and MET in the indicated breast cancer cells. TNBC, triple-negative breast cancer (*C*) MDA-MB-231 cells were treated with the indicated siRNAs and then stimulated with or without HGF (100 ng/ml) for 30 min. Cell lysates were subjected to immunoblot analysis for CDCP1 and MET. GAPDH was used as a loading control. *D*, MDA-MB-231 cells were treated with the indicated siRNAs and then stimulated with or without HGF (100 ng/ml) for the indicated time. Cell lysates were subjected to immunoblot analysis for CDCP1, p-MET, and MET. *E*, quantification of MET protein levels in the immunoblots shown in (*D*). *F*, quantification of p-MET levels in the immunoblots shown in (*D*). In (*E* and *F*), the mean ratios ± SD were obtained from three/four independent experiments. ∗*p* < 0.05; ∗∗*p* < 0.01; n.s., not significantly different; unpaired two-tailed *t* test.

To verify the importance of CDCP1 in HGF-induced invasion of human cancer cells (Fig. 1*A*), we used human triple-negative breast cancer cell lines, which highly expressed CDCP1 (19, 20) (Fig. 1*B*). Among these, we focused our analysis on the cells with the most abundant expression of MET, the metastatic MDA-MB-231 cell line. We first knocked down *CDCP1* using siRNA and examined its effects on HGF signaling (Fig. 1*C*). Immunoblot analysis showed that *CDCP1* knockdown significantly decreased MET protein levels even before HGF stimulation (Fig. 1, *C* and *E*). Consequently, MET activation was significantly attenuated in the initial phase of HGF signaling (Fig. 1, *D* and *F*). Since *CDCP1* knockdown did not affect *MET* mRNA expression (Fig. S1*A*), it is suggested that CDCP1 functionally interacts with MET and may contribute to the regulation of stability and/or turnover rate of MET protein in these cancer cells.

**Figure 2:**
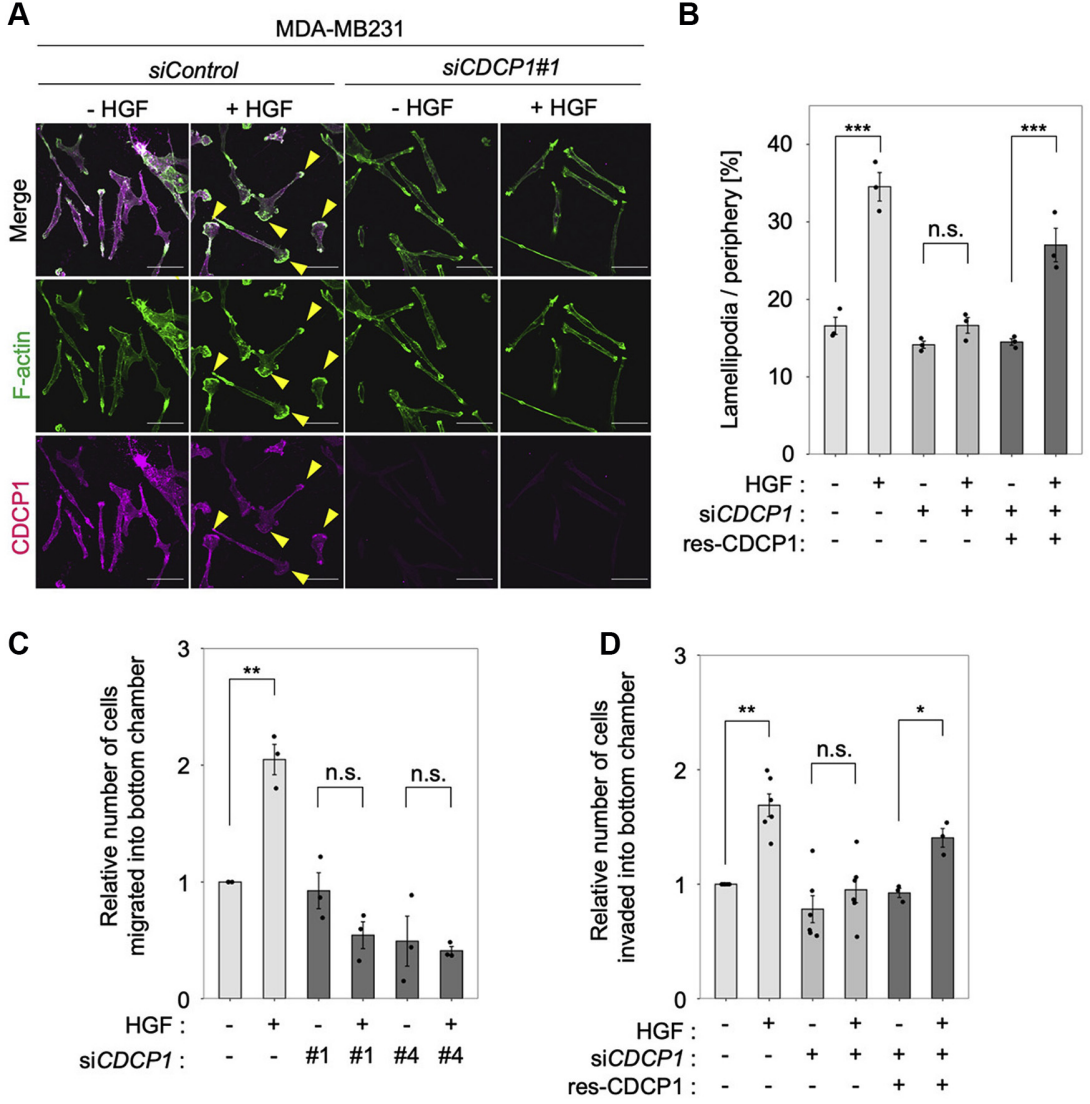
**CDCP1 is required for HGF-promoted cell migration and invasion in MDA-MB-231 cells.***A*, MDA-MB-231 cells were treated with the indicated siRNAs and then stimulated with or without HGF for 6 h. Cells were subjected to immunofluorescence staining for F-actin and CDCP1. *Yellow arrowheads* indicate lamellipodia. The scale bar represents 50 μm. *B*, ratio of the length of lamellipodia to that of total peripheral membrane. *C*, relative number of migrated cells. *D*, relative number of invaded cells. In (*B*–*D*), the mean ratios ± SD were obtained from three independent experiments. ∗*p* < 0.05; ∗∗*p* < 0.01; ∗∗∗*p* < 0.001; n.s., not significantly different; unpaired two-tailed *t* test.

We then investigated the role of CDCP1 in HGF-induced cell migration and invasion by observing the HGF-induced formation of the lamellipodium, an actin-enriched membrane structure at the leading edge of cells that functions to pull cells forward during migration (21). The HGF-induced formation of lamellipodia was inhibited by *CDCP1* knockdown and rescued by CDCP1 re-expression (Fig. 2, *A* and *B*). Of note, some CDCP1 coaccumulated with F-actin along the edge of lamellipodia in HGF-stimulated cells (Fig. 2*A*). Furthermore, Boyden chamber assays revealed that *CDCP1* knockdown significantly inhibited HGF-induced cell migration (Fig. 2*C*) and invasion (Fig. 2*D*). Furthermore, the inhibition of invasive activity was also rescued by CDCP1 re-expression (Fig. 2*D*). These observations suggest that CDCP1 is required for HGF-induced dynamic cell migration and invasion in MDA-MB-231 cells.

### CDCP1-SRC promotes HGF-induced cell invasion in human breast cancer cell line T47D

**Figure 3:**
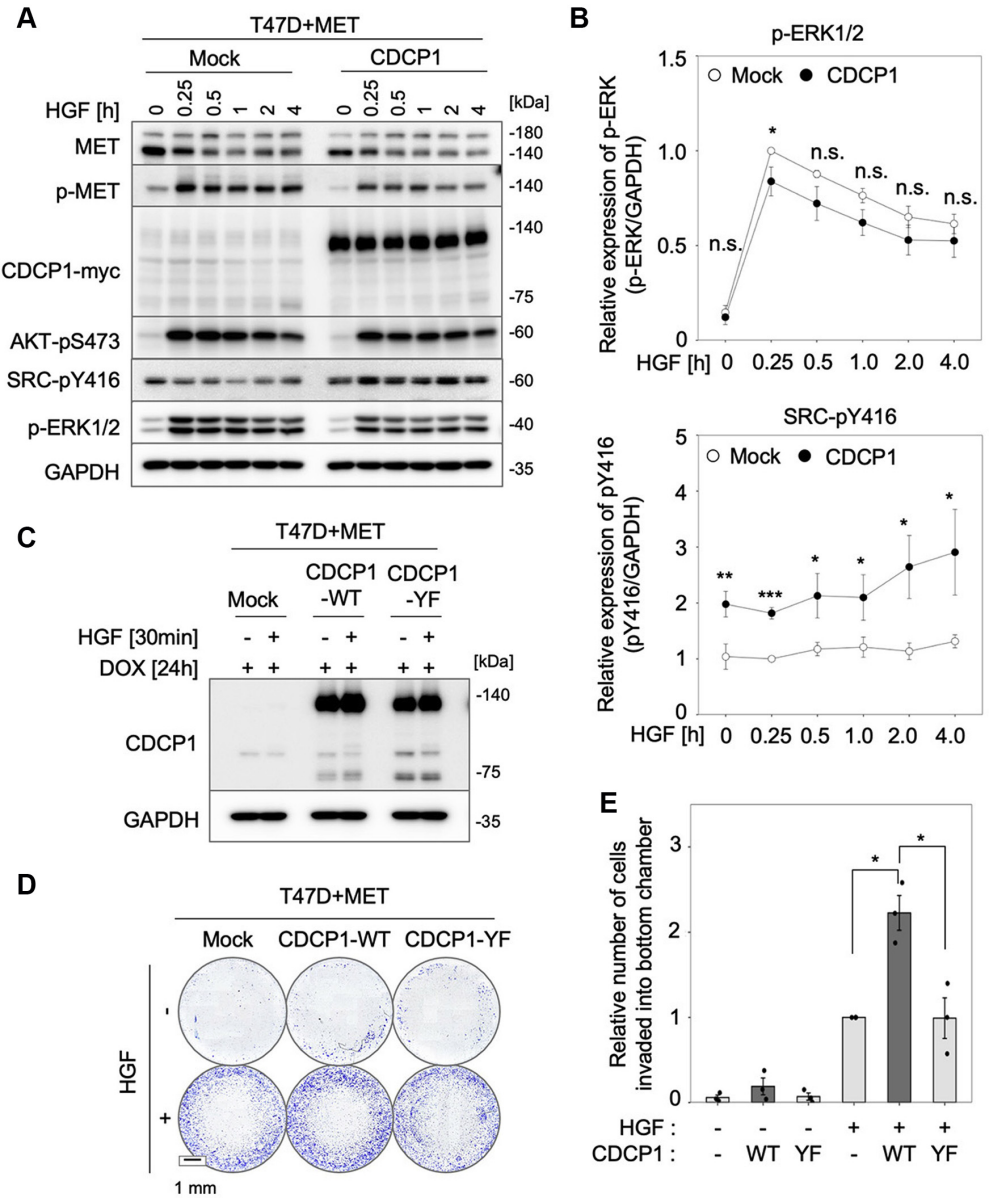
**CDCP1 promotes HGF-induced cell invasion in human breast cancer cell line T47D.***A*, T47D cells expressing MET with or without CDCP1 were stimulated with HGF for the indicated time, and cell lysates were subjected to immunoblot analysis for the indicated antigens. *B*, quantification of p-ERK1/2 and SRC-pY416 levels in the immunoblots shown in (*A*). *C*, immunoblot analysis for CDCP1 in T47D cells expressing MET with wild-type CDCP1 (CDCP1-WT) or a mutant CDCP1 that lacks SRC activation site (CDCP1-Y734F). *D*, the invasive activity of the above-mentioned cells was analyzed by Boyden chamber assay. Invaded cells on the lower surface of the chamber were stained with crystal violet. The scale bar represents 1 mm. *E*, relative number of invaded cells. In (*B* and *E*), the mean ratios ± SD were obtained from three/four independent experiments. ∗*p* < 0.05; ∗∗*p* < 0.01; ∗∗∗*p* < 0.001; n.s., not significantly different; unpaired two-tailed *t* test.

To further confirm the role of CDCP1 in HGF signaling, we subsequently employed a low invasive/nonmetastatic breast cancer cell line T47D, which had no significant expression of either protein (Fig. 1*B*) and no invasive activity (Fig. S1, *B* and *C*). We thus introduced MET into these cells using a Tet-On system and then established cell lines with or without stable CDCP1 overexpression. Effects of CDCP1 expression on HGF signaling were examined using immunoblot analysis (Fig. 3*A*). MET expression enabled these cells to respond to HGF stimulation, as indicated by the phosphorylation of MET, AKT, and ERK. Since MET was forcedly and continuously overexpressed in the distinct cell lines, the effects of CDCP1 expression on MET protein levels and cell signaling could not be accurately compared. Consequently, MET signaling (*e.g.*, p-ERK1/2) appeared unchanged or rather decreased by CDCP1 expression (Fig. 3, *A* and *B*). Nonetheless, CDCP1 expression induced a 2- to 3-fold increase in SRC-pY416 signals, indicating that SRC was selectively activated by CDCP1 expression (Fig. 3*B*). Under these conditions, the invasion assay using the Boyden chamber showed that HGF-induced invasive activity was enhanced by CDCP1 expression, but the expression of a mutant CDCP1 that lacks SRC activation site (CDCP1-Y734F) (12) failed to promote HGF-induced invasion (Fig. 3, *C*–*E*). These results suggest that the CDCP1-SRC axis is involved in the promotion of HGF-induced cell invasion.

### CDCP1-SRC promotes HGF-induced membrane ruffling in T47D cells by activating RAC1

**Figure 4:**
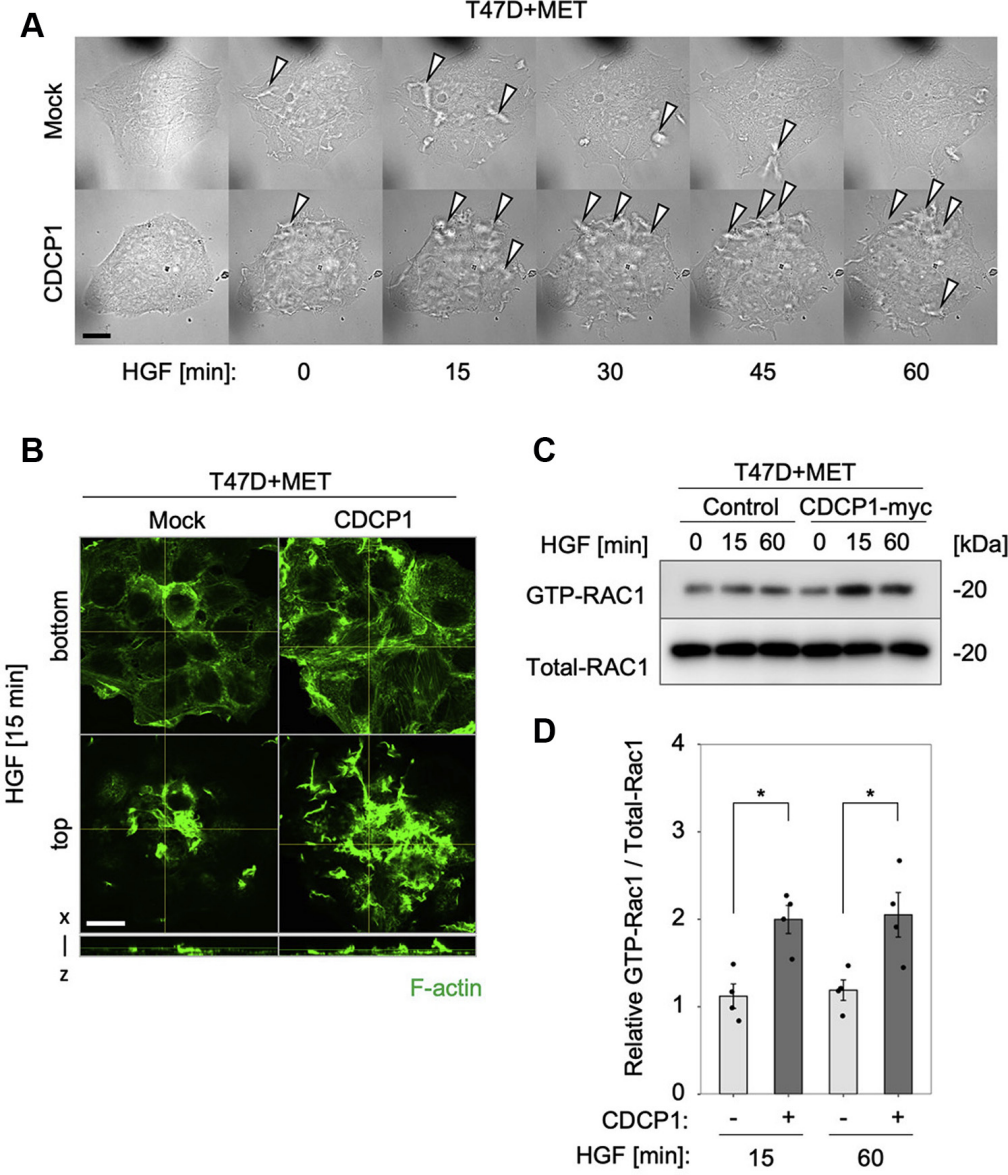
**CDCP1 promotes HGF-induced membrane ruffling in T47D cells.***A*, T47D cells expressing MET with or without CDCP1 were stimulated with HGF and observed under time-lapse microscopy. Phase contrast images at the indicated time after HGF stimulation are shown. Open arrowheads indicate a ruffled membrane. The scale bar represents 20 μm. Fluorescence images of Lifeact-GFP are shown in Movie S1–S4. *B*, T47D cells expressing MET with or without CDCP1-myc were stimulated with HGF for 15 min. The distribution of F-actin was analyzed by confocal microscopy. The bottom and top views are shown. The scale bar represents 20 μm. Depth of x–z, 10 μm (*C*) T47D cells expressing MET with or without CDCP1-myc were stimulated with HGF for the indicated time. The activity of RAC1 was determined by a pull-down assay, followed by immunoblot analysis for GTP-RAC1 and Total RAC1. *D*, signal intensities of the above blots. The mean ratios ± SD were obtained from four independent experiments. ∗*p* < 0.05; unpaired two-tailed *t* test.

Since T47D cells retained epithelial features, even though they overexpressed both MET and CDCP1, HGF stimulation was unable to promote lamellipodia formation. Thus, we observed effects of HGF stimulation on membrane dynamics in these cells. Time-lapse microscopy analysis revealed that CDCP1 expression dramatically enhanced the formation of membrane ruffles, which were enriched with the actin cytoskeleton (Movie S1–S4 and Fig. 4, *A* and *B*). Membrane ruffles and lamellipodia are known to be formed *via* RAC1-GTPase-mediated reorganization of the actin cytoskeleton (22). Inhibitor analysis in MET-expressing T47D cells indicated that CDCP1-dependent promotion of invasion activity was markedly suppressed by an inhibitor of RAC1 (Fig. S2*A*). The treatment with a phosphatidylinositol 3-kinase (PI3K) inhibitor LY294002 or a Src family kinase (SFK) inhibitor Dasatinib significantly inhibited the basal invasion activity, indicating the requirement of PI3K and SFK activities for promotion of cell invasion (Fig. S2, *A* and *B*). Furthermore, a pull-down assay for RAC1 activity showed that CDCP1 expression significantly enhanced HGF-dependent RAC1 activation (Fig. 4, *C* and *D*).

The roles of RAC1 and SRC in CDCP1-dependent promotion of HGF-induced cell invasion was further confirmed in MDA-MB-231 cells. HGF-induced lamellipodia formation was inhibited by RAC1 inhibitor but not by ROCK inhibitor Y27632 (Fig. S2*C*). The treatment with Dasatinib strongly suppressed cell invasion, and RAC1 inhibitor treatment suppressed HGF-dependent promotion of cell invasion (Fig. S2). Re-expression of wild-type CDCP1 significantly restored HGF-dependent invasive activity in *CDCP1* knockdown cells, whereas that of CDCP1-Y734F only partially rescued the phenotype (Fig. S2*D*). Furthermore, Dasatinib treatment significantly suppressed HGF-induced RAC1 activation (Fig. S2, *E* and *F*). Although the sensitivity to inhibitors was somehow different between the two cell lines, these data underscore the role of RAC1 and the CDCP1-SRC axis in HGF-induced invasion activity and raise a question of how the CDCP1-SRC axis activates RAC1.

### ARHGEF7 is critical for CDCP1-dependent promotion of cancer cell invasion

**Figure 5:**
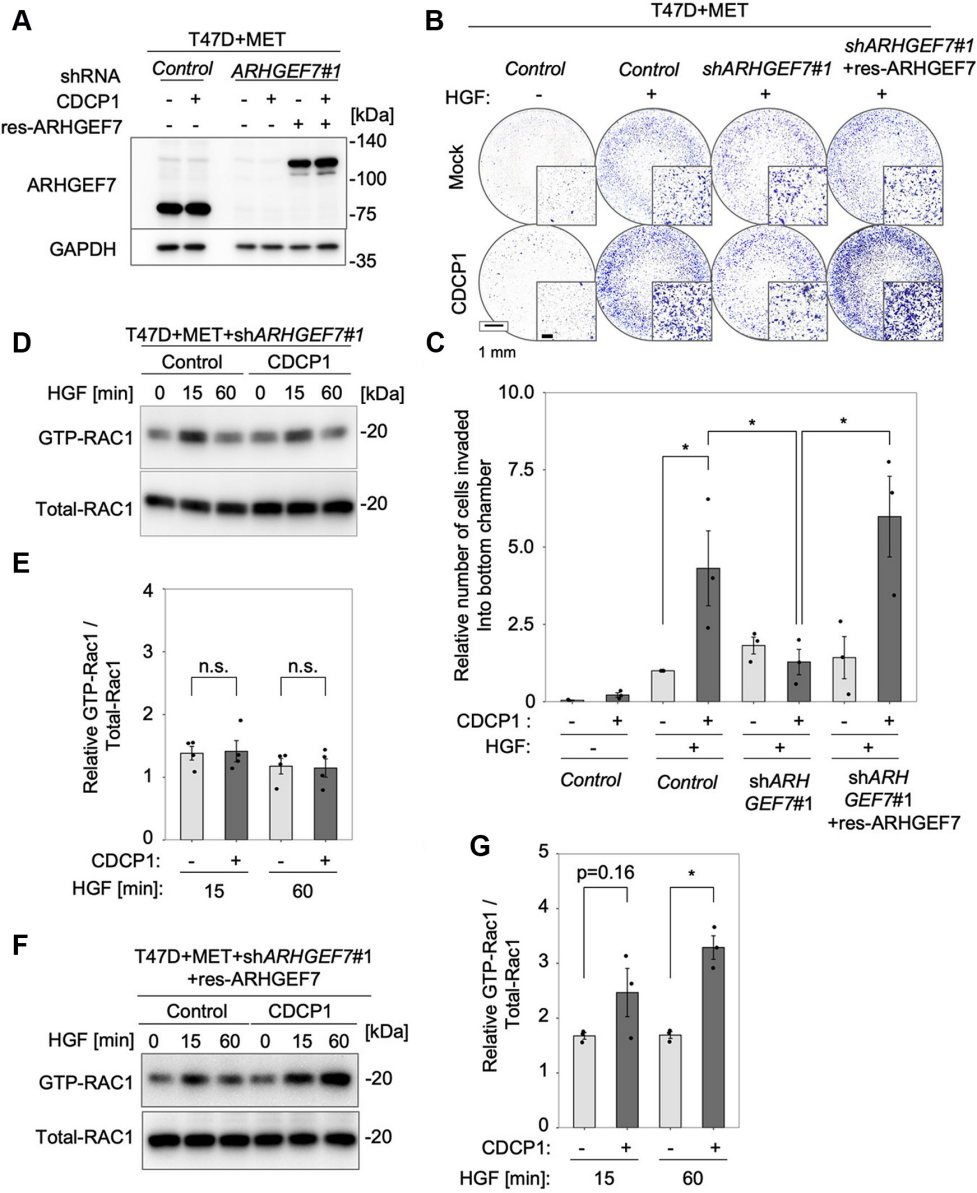
**ARHGEF7 is critical for CDCP1-dependent promotion of HGF-induced cell invasion in T47D cells expressing MET.***A*, T47D cells expressing MET with or without CDCP1-myc were transfected with *shControl* or *shARHGEF7#1*, and the cells treated with *shARHGEF7#1* were further transfected with or without shRNA-resistant ARHGEF7-mCherry construct (res-ARHGEF7). ARHGEF7 expression was confirmed by immunoblot analysis. *B*, T47D cell lines used in the above experiments were subjected to a Boyden chamber invasion assay. The scale bar represents 1 mm and 200 μm (*inset*). *C*, relative number of invaded cells. *D*, T47D cells expressing MET with or without CDCP1-myc were transfected with *shControl* or *shARHGEF7#1*. After HGF stimulation for the indicated time, the activity of RAC1 was determined by a pull-down assay. *E*, quantification of RAC1 activity. *F*, the cell lines used in (*D*) were further transfected with res-ARHGEF7 and analyzed for RAC1 activity. *G*, quantification of RAC1 activity. In (*C*, *E*, and *G*), the mean ratios ± SD were obtained from three/four independent experiments. ∗*p* < 0.05; n.s., not significantly different; unpaired two-tailed *t* test.

Since CDCP1 does not contain a structure that has the activity of guanine nucleotide exchange factor (GEF), we hypothesized that some RAC1 GEFs mediate the functional interplay between CDCP1 and RAC1. To explore this possibility, we knocked down several RAC1 GEFs, including ARHGEF7, VAV2, TIAM1, and DOCK1, which have been implicated in membrane ruffling (22, 23, 24, 25, 26) (Fig. S3*A*), and examined effects on CDCP1-dependent promotion of HGF-induced invasive activity in MET-expressing T47D cells (Fig. S3*B*). Among the GEFs tested, only *ARHGEF7* knockdown suppressed CDCP1-dependent activation of invasive activity. Knockdown of other GEFs suppressed the invasive activity independent of CDCP1. The rescue experiment using the shRNA-resistant ARHGEF7-mCherry construct (res-ARHGEF7) in MET-expressing T47D cells clearly revealed that ARHGEF7 re-expression restored CDCP1-dependent promotion of HGF-induced invasive activity (Fig. 5, *A*–*C*). Furthermore, *ARHGEF7* knockdown completely suppressed CDCP1-dependent RAC1 activation (Fig. 5, *D* and *E*), which was restored by res-ARHGEF7 expression (Fig. 5, *F* and *G*).

**Figure 6:**
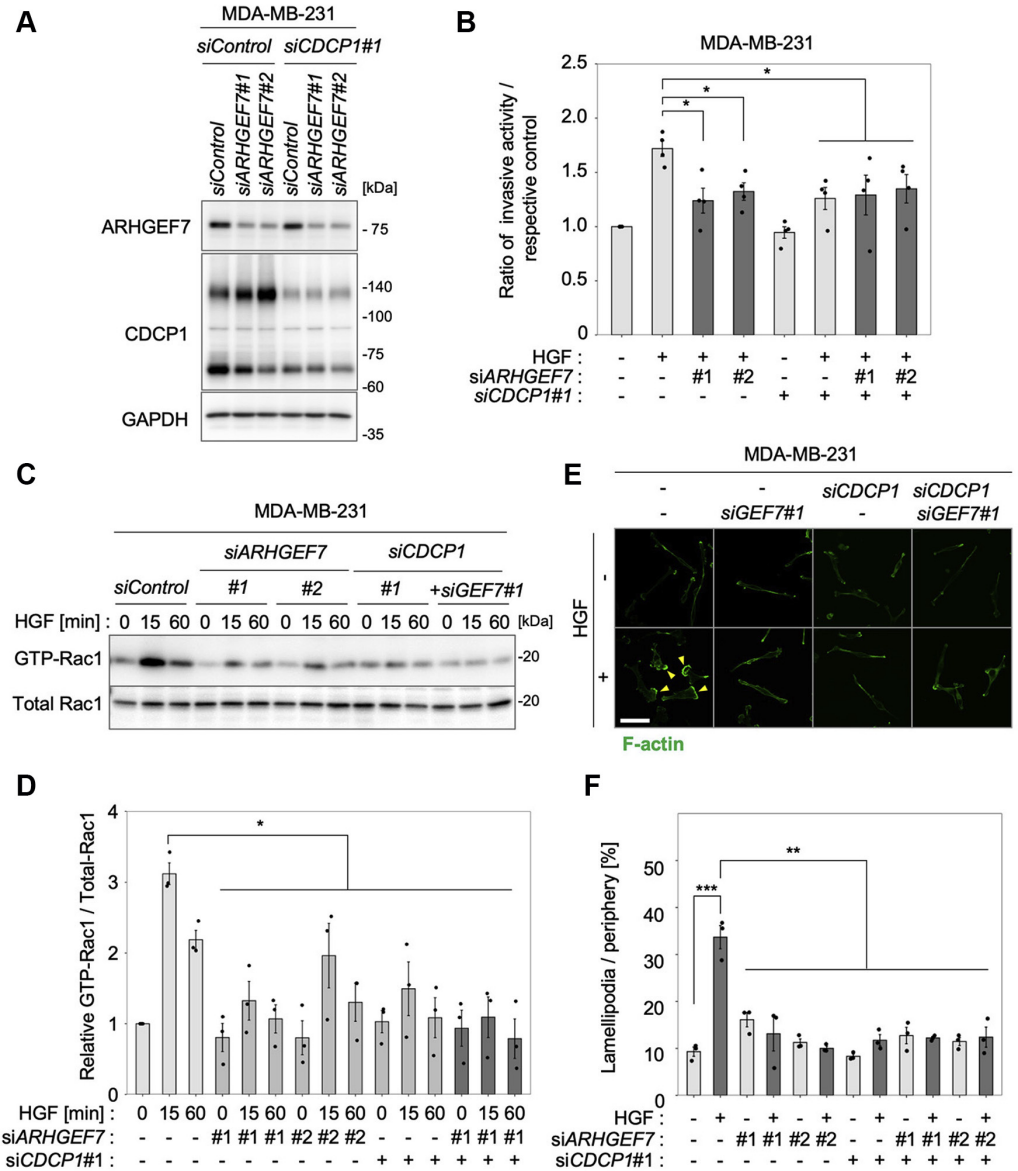
**ARHGEF7 is critical for CDCP1-dependent promotion of HGF-induced cell invasion in MDA-MB-231 cells.***A*, MDA-MB-231 cells were treated with the indicated siRNAs, and cell lysates were subjected to immunoblot analysis for ARHGEF7 and CDCP1 and MET. *B*, MDA-MB-231 cell lines used in the above experiments were subjected to a Boyden chamber invasion assay, and relative numbers of invaded cells were shown. *C*, MDA-MB-231 cell lines transfected with the indicated siRNAs were treated with HGF for the indicated time, and the activity of RAC1 was determined by a pull-down assay. *D*, quantification of RAC1 activity. *E*, MDA-MB-231 cells were treated with the indicated siRNA and then stimulated with HGF for 6 h. Cells were subjected to immunofluorescence staining for F-actin. *Yellow arrowheads* indicate lamellipodia. The scale bar represents 10 μm. *F*, ratio of the length of lamellipodia to that of total peripheral membrane. In (*B*, *D*, and *F*), the mean ratios ± SD were obtained from three independent experiments. ∗*p* < 0.05; ∗∗*p* < 0.01; ∗∗∗*p* < 0.001; unpaired two-tailed *t* test.

To validate the role of ARHGEF7, we knocked down *ARHGEF7* and/or *CDCP1* in MDA-MB-231 cells and examined their effects on HGF-induced invasive activity (Fig. 6). The results showed that ARHGEF7 was required for CDCP1-dependent promotion of HGF-induced invasive activity (Fig. 6, *A* and *B*) and other GEFs functioned independently of HGF signaling in these cells as well (Fig. S4, *A* and *B*). Furthermore, knockdown of *ARHGEF7* and/or *CDCP1* significantly suppressed HGF-induced RAC1 activation (Fig. 6, *C* and *D*) and lamellipodia formation (Fig. 6, *E* and *F*). Taking these findings together with the fact that SRC activity was required for HGF-induced RAC1 activation (Fig. S2, *E* and *F*), it is likely that HGF-induced RAC1 activation by CDCP1-SRC is preferentially dependent on ARHGEF7, raising the next question of how the CDCP1-SRC axis activates ARHGEF7.

### CDCP1 potentiates HGF-induced accumulation of ARHGEF7 on PIP3-enriched membrane domain

**Figure 7:**
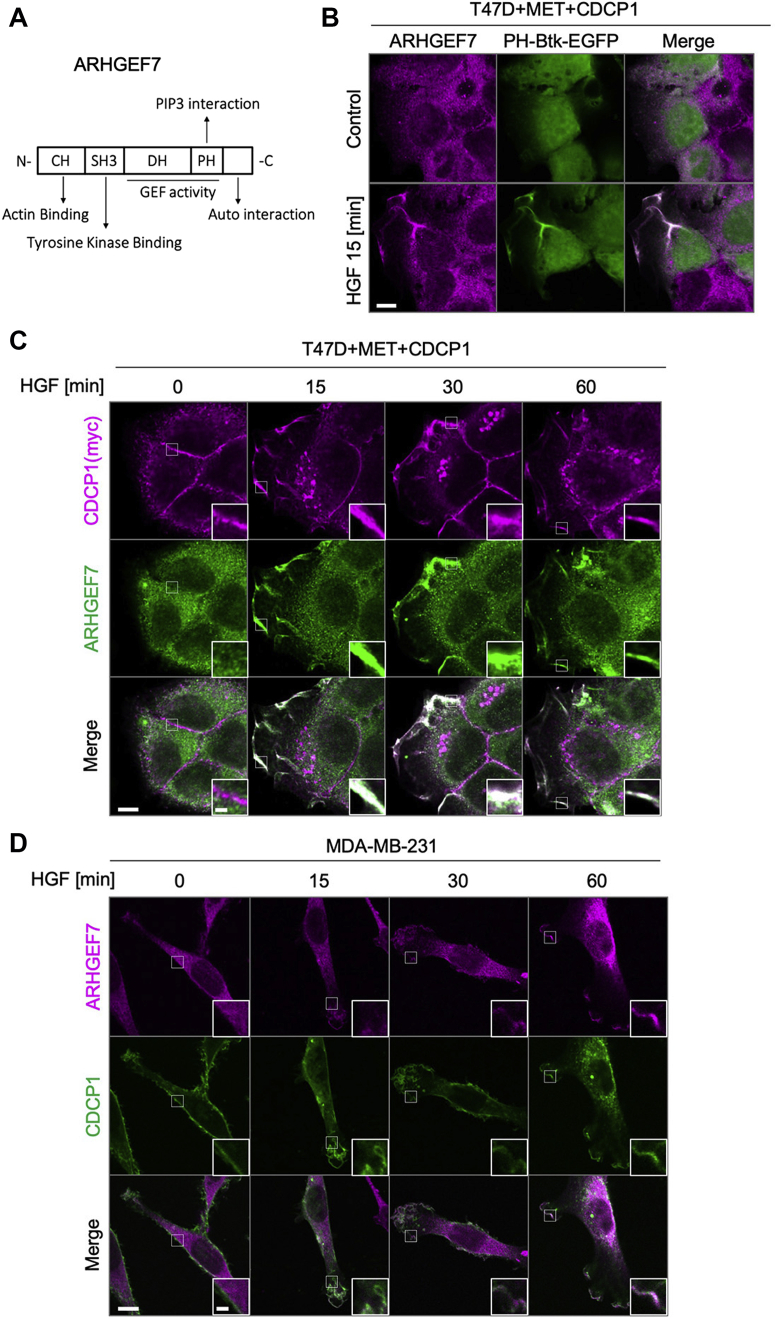
**CDCP1 potentiates HGF-induced accumulation of ARHGEF7 on the PIP3-enriched membrane domain.***A*, schematic diagram of ARHGEF7 structure. *B*, T47D cells expressing MET were transfected with the PH-Btk-EGFP construct and stimulated with or without HGF for 15 min. Subcellular localization of ARHGEF7 and PIP3 was visualized by immunofluorescence analysis. The scale bar represents 10 μm. *C*, T47D cells expressing MET and CDCP1 were stimulated with HGF for the indicated time and subjected to immunofluorescence staining for CDCP1 and ARHGEF7. The scale bar represents 10 μm and 2 μm (*inset*). *D*, MDA-MB-231 cells were stimulated with HGF for the indicated time and subjected to immunofluorescence staining for CDCP1 and ARHGEF7. The scale bar represents 10 μm and 2 μm (*inset*).

To elucidate the functional link between the CDCP1-SRC axis and ARHGEF7, we first examined the physical interactions between CDCP1 and ARHGEF7. However, a coimmunoprecipitation assay revealed that there was no stable interaction between these molecules. Furthermore, SRC-induced tyrosine phosphorylation of ARHGEF7 (27) was undetectable under our conditions. Thus, we investigated the subcellular localization of these molecules. Considering that ARHGEF7 has a PH domain (Fig. 7*A*), we hypothesized that ARHGEF7 can accumulate in the membrane region where PIP3 is enriched. Indeed, immunofluorescence analysis revealed that, upon HGF stimulation, ARHGEF7 accumulated in the membrane region where PH-Btk-EGFP, a PIP3 reporter (28, 29, 30), accumulated (Fig. 7*B*). Furthermore, HGF stimulation induced coaccumulation of ARHGEF7 with CDCP1 at the edge of cells where membrane ruffling occurred in T47D cells expressing MET and CDCP1 (Fig. 7*C*). The HGF-dependent coaccumulation of ARHGEF7 with CDCP1 was also observed at the edge of lamellipodia in MDA-MB-231 cells (Fig. 7*D*). Furthermore, HGF-induced coaccumulation of ARHGEF7 with CDCP1 was prevented by inhibiting MET (Fig. S5, *A* and *B*). Taking these observations together with the contribution of PI3K to HGF signaling (Fig. S2*A*), it is suggested that the CDCP1-SRC axis potentiates HGF-induced formation of the PIP3-enriched membrane domain, where CDCP1 and ARHGEF7 coaccumulate to activate RAC1-mediated membrane ruffling.

### Membrane localization of CDCP1 and MET is regulated *via* the endosome system

**Figure 8:**
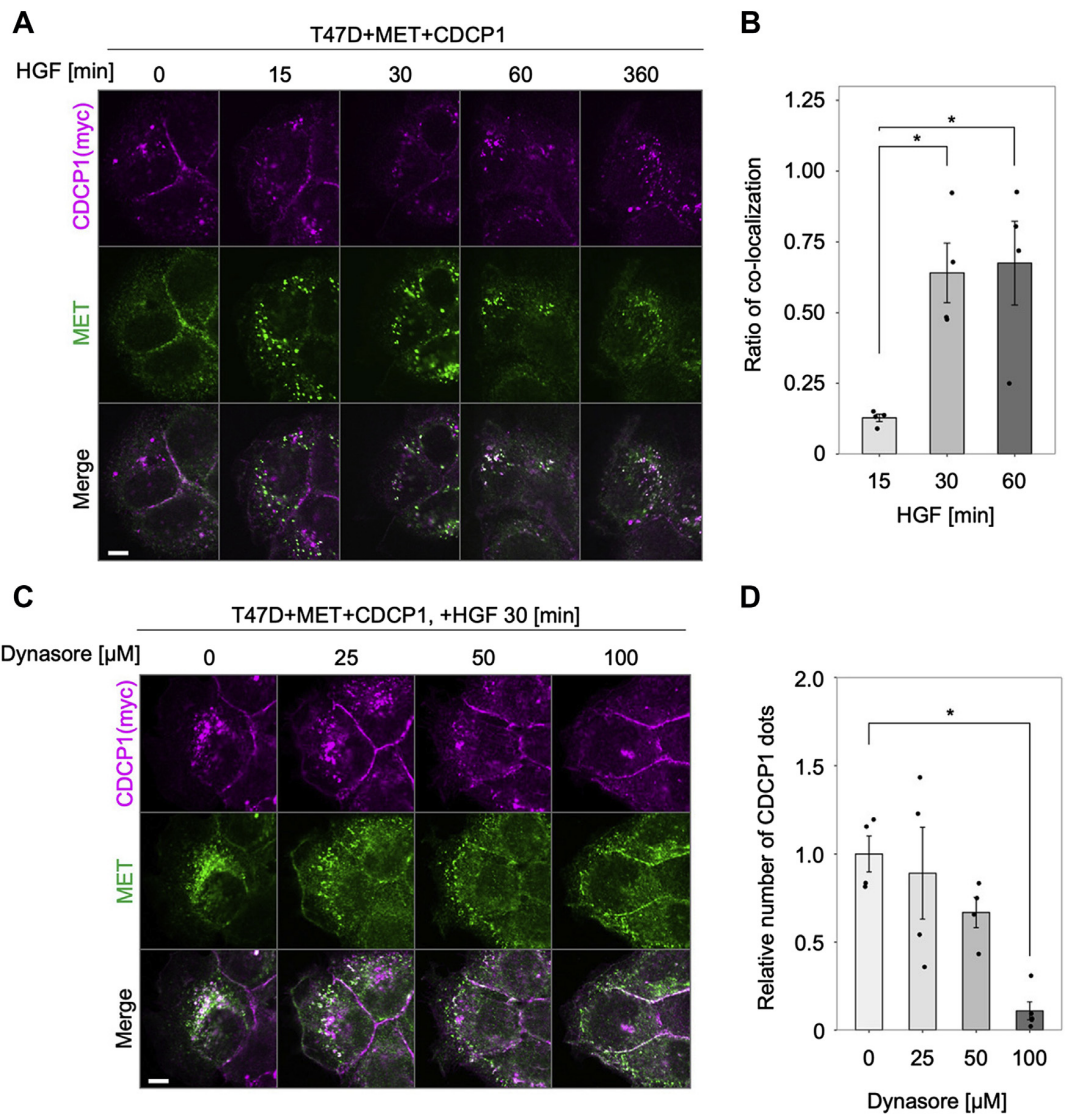
**Membrane localization of CDCP1 and MET is regulated *via* the endosome system.***A*, T47D cells expressing MET and CDCP1-myc were stimulated with HGF for the indicated time and subjected to immunofluorescence analysis for CDCP1-myc and MET. The scale bar represents 10 μm. *B*, ratios of colocalization of CDCP1 and MET were quantified. *C*, T47D cells expressing MET and CDCP1-myc were pretreated with Dynasore at the indicated concentration and stimulated with HGF for 30 min. Subcellular localization of CDCP1-myc and MET was analyzed by immunofluorescence analysis. The scale bar represents 10 μm. *D*, ratios of vesicle localization. In (*B* and *D*), the mean ratios ± SD were obtained from four independent experiments. ∗*p* < 0.05; unpaired two-tailed *t* test.

**Figure 9:**
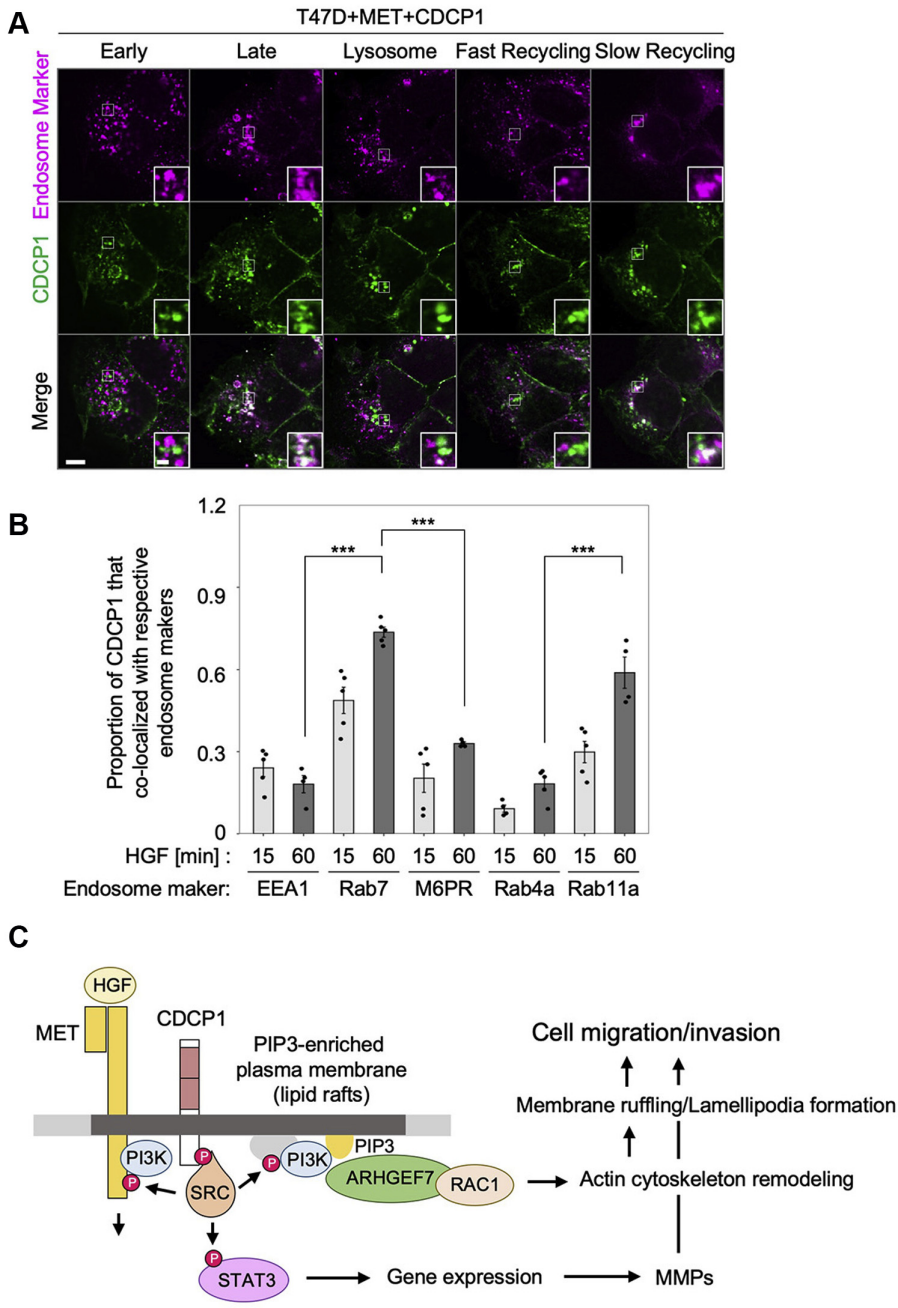
**CDCP1 is recycled *via* the endosome system.***A*, T47D cells expressing MET and CDCP1-myc were stimulated with HGF for 30 min and subjected to immunofluorescence analysis for CDCP1-myc and the indicated endosome markers. Insets are magnified views. The scale bar represents 10 μm and 2 μm (*inset*) *B*, ratios of CDCP1 colocalization with endosome markers. The mean ratios ± SD were obtained from five independent experiments. ∗∗∗*p* < 0.001; unpaired two-tailed *t* test. *C*, schematic model of our working hypothesis derived from this study.

Finally, we addressed how CDCP1 translocates to PIP3-enriched regions. To this end, we examined changes in the subcellular localization of CDCP1 and MET after HGF stimulation using immunofluorescence analysis (Fig. 8). Upon HGF stimulation, both proteins were rapidly endocytosed and colocalized on endosomes within 30 min after stimulation (Fig. 8, *A* and *B*). Pretreatment with Dynasore suppressed the endocytosis of both proteins, indicating that the process was clathrin dependent (Fig. 8, *C* and *D*). These observations suggest that CDCP1 interacts with MET during endocytosis. Furthermore, immunofluorescence analysis using endosome markers revealed that CDCP1 accumulated in Rab7-positive late endosomes, and a substantial part of CDCP1 was detected on Rab11a-positive slow recycling endosomes (Fig. 9, *A* and *B*). We also observed that tyrosine phosphorylation of CDCP1 gradually (∼60 min) increased concurrently with HGF-induced RAC1 activation and membrane ruffling (Fig. S6). These results suggest that activated CDCP1-SRC can be recycled back to the plasma membrane after HGF stimulation. Indeed, immunoblot analysis showed that CDCP1 protein levels were unchanged during HGF stimulation, while activated MET was rapidly degraded *via* lysosome or proteasome digestion (31) (Fig. 1*D*). These findings raise the possibility that activated CDCP1-SRC, which is selectively recycled back to the plasma membrane, contributes to forming the PIP3-enriched region where the ARHGEF7-RAC1 axis is activated.

## Discussion

We investigated the role of CDCP1 in human breast cancer cells and found that the CDCP1-SRC axis enhanced the HGF-induced formation of lamellipodia or membrane ruffles, leading to the promotion of cell migration and invasion. A mechanistic analysis revealed the ARHGEF7-RAC1 axis as a novel mediator of CDCP1-dependent promotion of cancer cell invasion. Furthermore, we showed that CDCP1 and ARHGEF7 coaccumulate *via* the endosome system on the HGF-induced PIP3-enriched membrane domain. These results led us to propose the following working hypothesis (Fig. 9*C*): HGF stimulation activates PI3K to induce a PIP3-enriched membrane domain, known as lipid rafts. Lipid raft-localized CDCP1 activates SRC, which can directly/indirectly promote the PI3K activation (32). CDCP1-SRC can also activate MET to potentiate HGF signaling (12). By linking these signaling circuits, CDCP1 may accelerate the local formation of PIP3-enriched signaling platform, where ARHGEF7 accumulates to activate RAC1-mediated membrane ruffling/lamellipodia formation. As reported previously (12), CDCP1 also contributes to the upregulation of gene expression required for invasive properties, such as matrix metalloprotease production, by activating the Src-STAT3 axis. Activating these multiple pathways promoted cell migration and invasion synergistically. This model sheds new light on the regulatory mechanism of HGF-MET signaling, which promotes cancer invasion and metastasis.

ARHGEF7, also known as Cool-1 and βPix, is an essential Dbl family of Rac GTPase GEF implicated in cytoskeleton remodeling, which is important in cell migration (33, 34, 35). In cancers, it has been shown that ARHGEF7 affects the motility of cancer cells *in vitro* and invasion *in vivo* (36, 37). Interesting, the ARHGEF7 gene is frequently amplified in metastatic lesions rather than at primary sites in colorectal adenocarcinoma (38). The Cancer Genome Atlas database analysis revealed that, although there is no significant correlation between CDCP1^high^ groups and poor prognoses in patients with breast cancer, ARHGEF7^high^ groups have a significant correlation with poor prognoses (Fig. S7, *A* and *B*). Furthermore, CDCP1^high^/ARHGEF7^high^ groups show stronger correlation with poor prognoses than other groups (Fig. S7*C*). These lines of evidence underscore the importance of ARHGEF7 in cancer malignancy. However, their regulatory mechanisms are not fully understood. In this study, we found that HGF-induced and CDCP1-dependent accumulation of ARHGEF7 onto the PIP3-enriched membrane domain triggers the activation of RAC1-mediated actin cytoskeleton remodeling, leading to the promotion of cell migration and invasion. Since various growth factors, such as EGF, can activate PIP3 production, it is likely that ARHGEF7 more widely contributes to cancer invasion/metastasis caused by amplification and/or mutation of various growth factor receptors.

We further addressed CDCP1 accumulation in PIP3-enriched membrane regions at the tips of membrane ruffles and lamellipodia. One explanation is that CDCP1 can be retained in PIP3-enriched lipid rafts *via* two palmitoylation moieties attached just underneath its transmembrane domain (12). Another possibility may be that CDCP1 can be dynamically trafficked *via* the endosome system. We found that CDCP1 was rapidly endocytosed following HGF stimulation and colocalized with MET at endosomes, where activation of the MET-CDCP1 axis occurs. Activated MET is downregulated *via* lysosomes or proteasomes, whereas CDCP1 is recycled back to the plasma membrane *via* recycling endosomes. The differential fates of MET and CDCP1 may be due to differences in their ubiquitination modes, which regulate the sorting of target proteins on the endosome membranes (39). Activated MET is selectively subjected to lysosomal or proteasomal digestion through ubiquitination (5, 31), although the fate determinant mechanism for CDCP1 remains unknown. Taking these pieces of information together, we propose that recycled CDCP1, which is concentrated and activated through the endosome system, may contribute to forming the PIP3-enriched region in the plasma membrane where the ARHGEF7-RAC1 axis is activated. Since CDCP1 can functionally interact with other transmembrane receptors, including HER2, EGFR, and integrin β1 (40, 41), it is possible that CDCP1 is involved in regulating receptor signaling in a manner similar to that of MET.

In conclusion, our studies in breast cancer cell lines identified the CDCP1-SRC-ARHGEF7-RAC1 axis as a crucial mediator of HGF-induced cancer cell migration and invasion. Further extensive investigation of this axis could provide new clues for understanding the mechanism of cell invasion and metastasis induced by various growth factors implicated in cancer malignancy.

## Experimental procedures

### Cell culture

MDA-MB-231, T47D, and Hs578T cells were cultured in Dulbecco's modified Eagle medium containing 10% fetal bovine serum (FBS) at 37 °C with 5% CO_2_. BT-549 and HCC-1937 cells were cultured in RPMI1640 medium containing 10% FBS at 37 °C with 5% CO_2_. For experiments with HGF stimulation, cells were plated 48 h before HGF treatment. Cells were then serum starved, and 1 mg/ml doxycycline was added for 24 h before adding 100 ng/ml HGF to serum-free medium for varying times. Recombinant human HGF (insect derived) was purchased from PeproTech.

### Antibodies and inhibitor

The primary antibodies used in this study were Myc-Tag (9B11), CDCP1, Met (D1C2), phospho-Met (Tyr1234/1235) (D26), phospho-tyrosine (P-Tyr-1000), phospho-Src (Tyr416), phospho-Akt (Ser473), phospho-p44/42 MAPK (Erk1/2) (Thr202/Tyr204), Vav2 (C64H2), and Rab7 (D95F2) obtained from Cell Signaling Technology. Anti-GAPDH antibody was purchased from Santa Cruz Biotechnology. Anti-EEA1 and Anti-Rac1 antibodies were purchased from BD Biosciences. Anti-M6PR antibodies were purchased from Thermo Fisher Scientific. Anti-ARHGEF7 antibody was prepared by immunizing rabbits with C-terminal fragments. Dynamin inhibitor Dynasore, PI3K inhibitor LY294002, and SFK inhibitor Dasatinib were purchased from Abcam. ROCK inhibitor Y27632 was purchased from Cayman chemical. RAC1 inhibitor was purchased from Merk. MET inhibitors, AMG337 and Crizotinib, were purchased from Selleck Chemicals.

### Immunoblotting and immunoprecipitation

For Western blotting and immunoprecipitation assays, cells were lysed with RIPA buffer (25 mM Tris-HCl [pH 7.6], 150 mM NaCl, 1% [*v*/*v*] NP-40, 0.1% [*w*/*v*] SDS, 0.5 mM EDTA, 0.25 mM EGTA, 1% (*w*/*v*) sodium deoxycholate, 1 mM Na_3_VO_4_, 20 mM NaF, 1 mM phenylmethylsulfonyl fluoride, and protease inhibitor cocktail) (Nacalai Tesque). For the immunoprecipitation assay, cell lysates were incubated with antibodies at 4 °C. Immunoprecipitated proteins were then pulled down using protein G-Sepharose (GE Healthcare). Horseradish peroxidase–conjugated anti-mouse or anti-rabbit IgG (Zymed Laboratories Inc) was used as the secondary antibody. All immunoblots were visualized and quantitated using a Luminograph II System (Atto).

### Immunofluorescence microscopy

For two-dimensional culture, cells were grown on coverslips coated with type-I collagen, fixed with 4% paraformaldehyde (PFA), and permeabilized with phosphate-buffered saline (PBS) containing 0.03% Triton X-100. For three-dimensional culture, cysts embedded within the collagen matrix were fixed with 4% PFA and permeabilized with PBS containing 0.5% Triton X-100. Permeabilized cells and cysts were blocked with 1% bovine serum albumin and incubated with primary antibodies, followed by incubation with Alexa Fluor 488/594-phalloidin (Molecular Probes). Immunostained objects were observed using an FV1000 confocal microscope (Olympus Corporation). For time-lapse observations, cells were plated on a glass-bottom dish (IWAKI) and observed using a Ti-E inverted microscope (Nikon).

### Plasmid construction and gene transfer

cDNA of CDCP1, siRNA-resistant CDCP1, Rab4a, Rab11a, ARHGEF7, shRNA-resistant ARHGEF7, and the PH domain of Btk were generated by PCR using human cDNA as the template and subcloned into the pCX4 retroviral plasmid (generously donated by Dr Akagi). siRNA-resistant CDCP1 and shRNA-resistant ARHGEF7 were generated by mutagenesis PCR. shRNAs against GEFs-mRNA were generated using PCR and a pLKO.1-shRNA vector and subsequently introduced by lentiviral infection. Detailed information on the primers is provided in Supporting information Table S1. All PCR experiments were performed using KOD-Plus polymerase (Toyobo Co, Ltd). Primer sequences used for mutagenesis and generation of shRNAs are listed in Supporting information Table S2. MET was subcloned into the pRetroX-TRE3G retroviral plasmid (Clontech Laboratories). All constructs were confirmed by sequencing. Gene transfer of pCX4 and pRrtroX-TRE3G was carried out by retroviral infection. Retroviral production was performed using Plat-E cells. Lipofection of viral vectors into Plat-E cells was performed using PEI MAX (Polysciences Inc). siRNAs were purchased from Sigma-Aldrich (St Louis) and transfected with Lipofectamine RNAiMAX (Thermo Fisher Scientific). siRNAs used are listed in Supporting information Table S3.

### qPCR assay

RNA from MDA-MB-231 was collected with NucleoSpin RNA Plus (MACHEREY-NAGEL). Then 1 μg of Collected RNA samples was used for RT-PCR with ReverTra Ace qPCR Mix (Toyobo Co, Ltd). qPCR assays were performed with QnantStudio 5 (Thermo Fisher Scientific) and THUNDERBIRD NEXT SYBR qPCR Mix (Toyobo Co, Ltd).

The primers used in *MET* mRNA quantification are

Forward: ACCTTTGATATAACTGTTTACTTGTTGCA,

Reverse: GCTTTAGGGTGCCAGCATTTTAG.

The primers used in *GAPDH* mRNA quantification are

Forward: GCTCTCTGCTCCTCCTGTTC,

Reverse: CGCCCAATACGACCAAATCC.

### GTP-Rac1 pull-down assay

PAK-PBD beads were purchased from Cytoskeleton Inc. Cells were lysed using Rac1 IP buffer (50 mM Tris-HCl [pH 7.5], 10 mM MgCl_2_, 0.5 M NaCl, and 2% Nonidet P-40), and the protein concentration was adjusted to 0.5 mg/ml. For the pull-down assay, 800 μl of cell lysate was used for each time point, and 10 μl of PAK-PBD beads was added to each lysate. Bound GTP-Rac1 was detected by Western blotting using an anti-Rac1 antibody (BD Biosciences).

### *In vitro* migration and invasion assay

BioCoat cell culture permeable supports and Matrigel Invasion Chambers (Corning Inc) were used for the migration and invasion assays, respectively. Cells (0.5 × 10^5^ for migration assays and 1 × 10^5^ for invasion assays) were seeded on inserts and transferred to chambers containing culture media and 0.1% FBS, with or without 100 ng/ml HGF. After incubation at 37 °C for 24 h, migrated or invaded cells were fixed with 4% PFA and stained with 1% crystal violet. Invasive cells were counted. Migration and invasion assays were repeated thrice.

### Clinical and gene expression analysis

Clinical and RNA sequencing data of breast cancer (1209 patients) from The Cancer Genome Atlas dataset were used. Survival curves were constructed using the Kaplan–Meier method and compared by using the log-rank test.

### Statistics and reproducibility

For data analyses, unpaired two-tailed *t* tests were performed to determine differences between groups. A two-way analysis of variance (ANOVA) was used for multiple group comparisons. A *p*-value of <0.05 was considered statistically significant. All data and statistics were derived from at least three independent experiments.

## Data availability

All data are contained within the article.

## Supporting information

This article contains supporting information.

## Conflict of interest

The authors declare that they have no conflicts of interest with the contents of this article.
