# Supplementary material for: SRC kinase activator CDCP1 promotes hepatocyte growth factor–induced cell migration/invasion of a subset of breast cancer cells
Source: J Biol Chem. 2022 Jan 24;298(3):101630. doi: 10.1016/j.jbc.2022.101630 (PMC8867115; doi:10.1016/j.jbc.2022.101630)
Supplement: Supplemental Figures S1–S7 and Tables S1–S3 [file mmc5.pdf]

Supporting information

SRC kinase activator CDCP1 promotes hepatocyte growth factor-induced cell migration/invasion of a subset of breast cancer cells

Naoyuki Kawase, Atsuya Sugihara, Kentaro Kajiwara, Michio Hiroshima, Kanako Akamatsu, Shigeyuki Nada, Kunio Matsumoto, Masahiro Ueda and Masato Okada

Supplementary Movie 1-4

Movie 1 and 2. Time-lapse microscopy analysis MET-expressing T47D cells.

Fluorescence images of Lifeact-GFP were observed for 1h after HGF stimulation.

Movie 3 and 4. Time-lapse microscopy analysis MET-expressing T47D cells with CDCP1 expression.

Fluorescence images of Lifeact-GFP were observed for 1h after HGF stimulation.

Supplementary Table 1-3

Supplementary Fig. S1-S7

Supplementary Table. S1. List of primers for shRNAs.

shARHGEF7#1	Forward sequence:	5’-CCGGCTCTGCTACAAGGAGGATCTTCTCGAGAAGATCCTCCTTGTAGCAGAGTTTTTG-3’
	Reverse sequence:	5’-AATTCAAAAACTCTGCTACAAGGAGGATCTTCTCGAGAAGATCCTCCTTGTAGCAGAG-3’
shARHGEF7#2	Forward sequence:	5’-CCGGGAAGTTAAGTTCAGCAAACATCTCGAGATGTTTGCTGAACTTAACTTCTTTTTG-3’
	Reverse sequence:	5’-AATTCAAAAAGAAGTTAAGTTCAGCAAACATCTCGAGATGTTTGCTGAACTTAACTTC-3’
shARHGEF7#3	Forward sequence:	5’-CCGGGCGGATATTAGTGTCGTGCAACTCGAGTTGCACGACACTAATATCCGCTTTTTG-3’
	Reverse sequence:	5’-AATTCAAAAAGCGGATATTAGTGTCGTGCAACTCGAGTTGCACGACACTAATATCCGC-3’
shARHGEF7#4	Forward sequence:	5’-CCGGGCCCTCCCAAAGGATTTGATACTCGAGTATCAAATCCTTTGGGAGGGCTTTTTG-3’
	Reverse sequence:	5’-AATTCAAAAAGCCCTCCCAAAGGATTTGATACTCGAGTATCAAATCCTTTGGGAGGGC-3’
shVAV2#1	Forward sequence:	5’-CCGGGCCACGATAAATTTGGATTAACTCGAGTTAATCCAAATTTATCGTGGCTTTTTG-3’
	Reverse sequence:	5’-AATTCAAAAAGCCACGATAAATTTGGATTAACTCGAGTTAATCCAAATTTATCGTGGC-3’
shVAV2#2	Forward sequence:	5’-CCGGACAGCATCGCGCAGAACAAAGCTCGAGCTTTGTTCTGCGCGATGCTGTTTTTTG-3’
	Reverse sequence:	5’-AATTCAAAAAACAGCATCGCGCAGAACAAAGCTCGAGCTTTGTTCTGCGCGATGCTGT-3’
shVAV2#3	Forward sequence:	5’-CCGGGTGGACAAGACTCGCAGATTTCTCGAGAAATCTGCGAGTCTTGTCCACTTTTTG-3’
	Reverse sequence:	5’-AATTCAAAAAGTGGACAAGACTCGCAGATTTCTCGAGAAATCTGCGAGTCTTGTCCAC-3’
shTiam#1	Forward sequence:	5’-CCGGACAACCCTGACTGCGACATTTCTCGAGAAATGTCGCAGTCAGGGTTGTTTTTTG-3’
	Reverse sequence:	5’-AATTCAAAAAACAACCCTGACTGCGACATTTCTCGAGAAATGTCGCAGTCAGGGTTGT-3’
shTiam#2	Forward sequence:	5’-CCGGCGCACCTACGTGAAGGATTTACTCGAGTAAATCCTTCACGTAGGTGCGTTTTTG-3’
	Reverse sequence:	5’-AATTCAAAAACGCACCTACGTGAAGGATTTACTCGAGTAAATCCTTCACGTAGGTGCG-3’
shTiam#3	Forward sequence:	5’-CCGGTTCGAAGGCTGTACGTGAATACTCGAGTATTCACGTACAGCCTTCGAATTTTTG-3’
	Reverse sequence:	5’-AATTCAAAAATTCGAAGGCTGTACGTGAATACTCGAGTATTCACGTACAGCCTTCGAA-3’
shDOCK1#1	Forward sequence:	5’-CCGGCCTTAACAAGTACGGAGATATCTCGAGATATCTCCGTACTTGTTAAGGTTTTTG-3’
	Reverse sequence:	5’-AATTCAAAAACCTTAACAAGTACGGAGATATCTCGAGATATCTCCGTACTTGTTAAGG-3’
shDOCK1#2	Forward sequence:	5’-CCGGCGTGGCAGATTACGGGAATTTCTCGAGAAATTCCCGTAATCTGCCACGTTTTTG-3’
	Reverse sequence:	5’-AATTCAAAAACGTGGCAGATTACGGGAATTTCTCGAGAAATTCCCGTAATCTGCCACG-3’
shDOCK1#3	Forward sequence:	5’-CCGGAGCACGATCTTATCGTCTATACTCGAGTATAGACGATAAGATCGTGCTTTTTTG-3’
	Reverse sequence:	5’-AATTCAAAAAAGCACGATCTTATCGTCTATACTCGAGTATAGACGATAAGATCGTGCT-3’

Supplementary Table. S2. List of primers for shRNA-resistant ARHGEF7.

mutagenesis-1st-res-shARHGEF7#1	Forward sequence:	ctctctgctataaagaggatcttagtaagagccc
	Reverse sequence:	aagatcctctttatagcagagagcagctgagg
mutagenesis-2nd-res-shARHGEF7#1	Forward sequence:	gctgctctatgttataaagaggatcttagtaag
	Reverse sequence:	ctctttataacatagagcagctgagggccgg
mutagenesis-1st-res-shARHGEF7#2	Forward sequence:	gtgagaagttgagctcagcaaacatttcatatttaatggg
	Reverse sequence:	gtttgctgagctcaacttctcactggtctgcaatgg
mutagenesis-2nd-res-shARHGEF7#2	Forward sequence:	gttgagctcggcgaacatttcatatttaatgggaaatc
	Reverse sequence:	tgaaatgttcgccgagctcaacttctcactggtc

Supplementary Table. S3. List of siRNAs.

	MISSION siRNA ID
siCDCP1#1	SASI_Hs01_00047185
siCDCP1#4	SASI_Hs01_00047188
siARHGEF7#1	SASI_Hs02_00325961
siARHGEF7#2	SASI_Hs02_00325962
siVAV2#1	SASI_Hs01_00118656
siVAV2#2	SASI_Hs01_00118657
siVAV2#3	SASI_Hs01_00118658
siTIAM1#1	SASI_Hs01_00139463
siTIAM1#2	SASI_Hs01_00139464
siTIAM1#3	SASI_Hs01_00139465
siDOCK1#1	SASI_Hs01_00246616
siDOCK1#2	SASI_Hs01_00246617
siDOCK1#3	SASI_Hs01_00246618

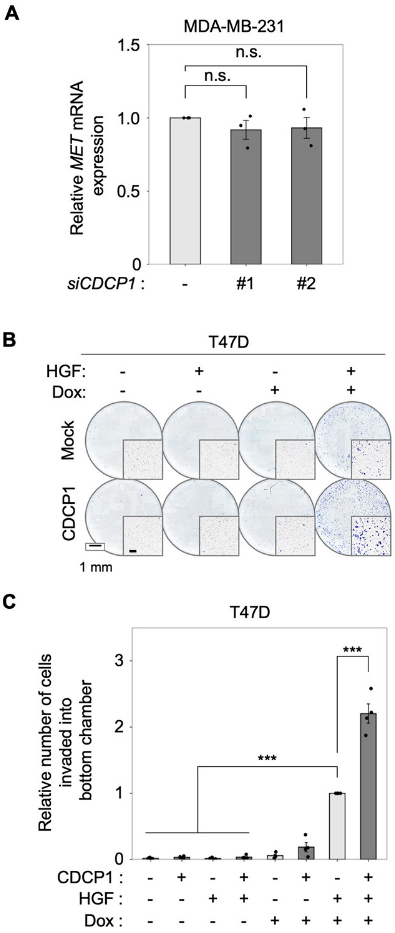

Fig.S1. (A) *CDCP1* knockdown does not affect the expression of *MET* mRNA in MDA-MB-231 cells. MET mRNA levels in MDA-MB-231 cells treated with or without *siCDCP1* were determined by real-time PCR. **(B) T47D cells do not have invasive activity without MET expression.** T47D cells treated with or without HGF or Dox were subjected to a Boyden chamber invasion assay. Scale bar, 1 mm and 200 µm (inset). **(C)** Relative number of invaded cells. In **(A and C)**, the mean ratios ± SD were obtained from three independent experiments. ***, $P < 0.001$; n.s., not significantly different; Unpaired two-tailed *t*-test.

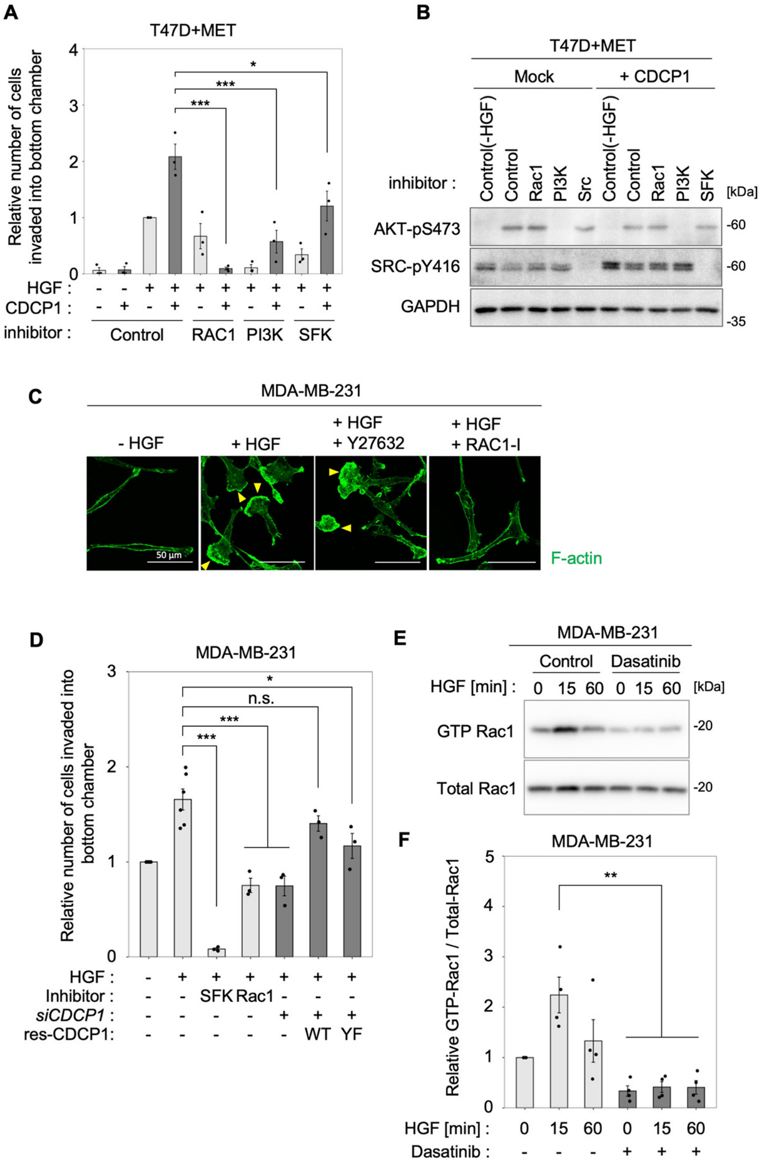

Fig.S2. (A) HGF-induced cell invasion in MET-expressing T47D cells is suppressed by an inhibitor of RAC1, PI3K, or SFK. T47D cells expressing MET with or without CDCP1 were stimulated with HGF in the presence or absence of the indicated inhibitors (RAC1 inhibitor; 100 μM, PI3K inhibitor LY294002; 10 μM, SFK inhibitor Dasatinib; 0.1 μM), and the invasive activity was analyzed by Boyden chamber assay, and relative number of invaded cells was shown. **(B)** Effects of inhibitors were confirmed by immunoblot analysis. Total lysates prepared from the cells used in above experiments were subjected to immunoblot analysis for the indicated phosphoproteins. **(C) Lamellipodia formation in MDA-MB-231 is inhibited by RAC1 inhibitor.** MDA-MB-231 cells were pre-treated with or without the indicated inhibitors (Y2763; 10 μM, RAC1 inhibitor; 100 μM) and then stimulated with HGF for 6 h. Cells were subjected to immunofluorescence staining for F-actin. Yellow arrowheads indicate lamellipodia. Scale bar, 50 μm. **(D) Invasive activity of MDA-MB-231 cells is suppressed by inhibiting RAC1 and SRC activity.** MDA-MB-231 cells were pre-treated with or without the indicated inhibitors or transfected with siCDCP1 together with or without wild-type res-CDCP1 (WT) or res-CDCP1-Y734F (YF), and subjected to Boyden chamber assay in the absence or presence of HGF stimulation. (**E**) **RAC1 activity in MDA-MB-231 cells is suppressed by inhibiting SRC activity.** MDA-MB-231 cells were pre-treated with or without Dasatinib. After HGF stimulation for the indicated time, the activity of RAC1 was determined by a pull-down assay. (**F**) Quantification of the RAC1 activity. In (**A**, D, **E**), the mean ratios ± SD were obtained from three/four independent experiments. *, $P < 0.05$; **, $P < 0.01$; ***, $P < 0.001$; n.s., not significantly different; Unpaired two-tailed *t*-test.

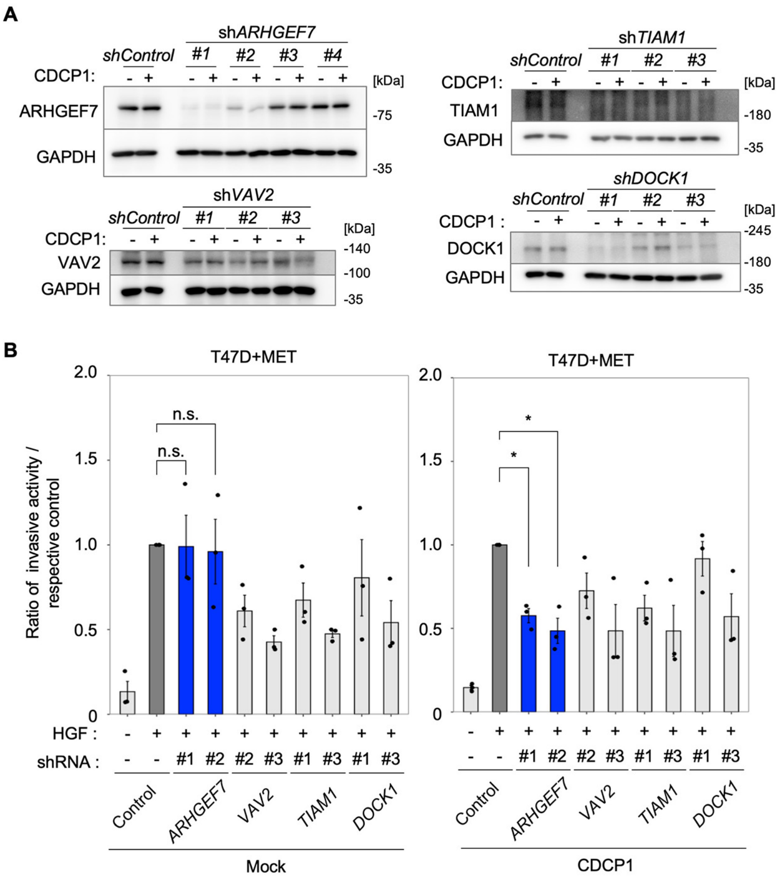

Fig.S3. Identification of GEFs responsible for CDCP1-dependent promotion of cell invasion in T47D cells expressing MET.

(A) T47D cells expressing MET with or without CDCP1 were transfected with the indicated shRNAs, and cell lysates were subjected to immunoblot analysis for the indicated GEF proteins. **(B)** T47D cells expressing MET with or without CDCP1 were transfected with the indicated shRNAs, and the invasive activity was determined using the Boyden chamber assay in the presence of HGF. Ratios of invasive activity were quantified. HGF-induced activity in *shControl*-transfected cells is defined as 1 in Mock and CDCP1 expressing cells. The mean ratios ± SD were obtained from three independent experiments. *, $P < 0.05$; n.s., not significantly different; Unpaired two-tailed t-test.

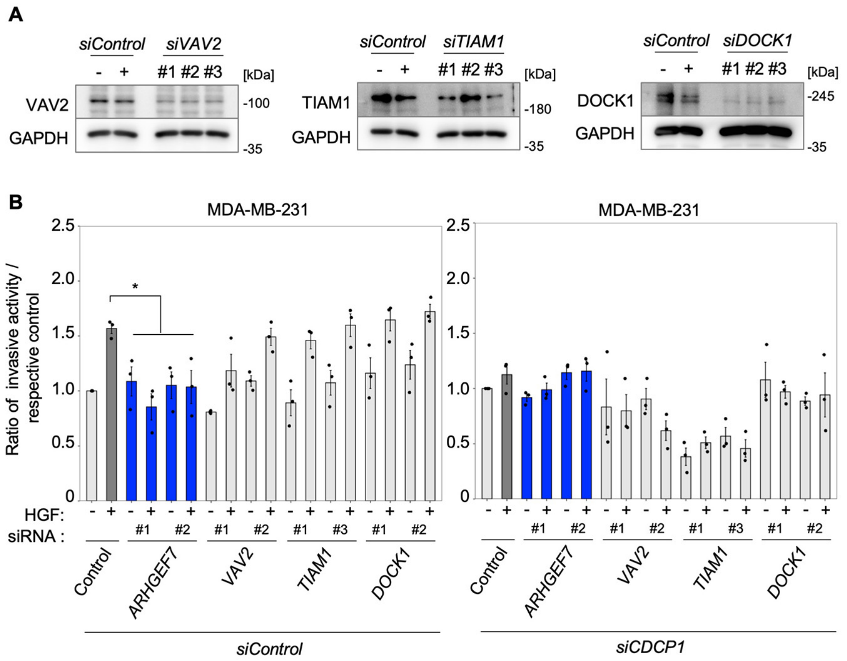

Fig.S4. Identification of GEFs responsible for CDCP1-dependent promotion of cell invasion in MDA-MB-231cells.

(A) MDA-MB-231 cells were transfected with the indicated siRNAs, and cell lysates were subjected to immunoblot analysis for the indicated GEF proteins. **(B)** MDA-MB-231 cells were transfected with the indicated siRNAs, and the invasive activity was determined using the Boyden chamber assay in the presence or absence of HGF. Ratios of invasive activity were quantified. The mean ratios ± SD were obtained from three independent experiments. *, $P < 0.05$; Unpaired two-tailed *t*-test.

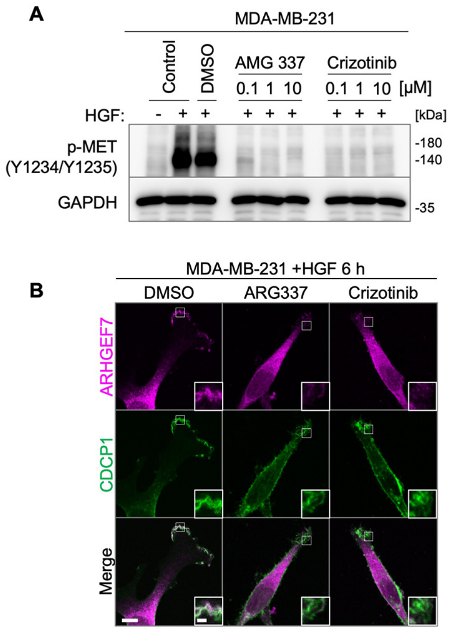

Fig.S5. Effects of MET inhibition on co-accumulation of CDCP1 and ARHGEF7 in MDA-MB-231cells.

(A) MDA-MB-231 cells pre-treated with MET inhibitor (AMG337 or Crizotinib) at the indicated concentrations were stimulated with or without HGF for 30 min, and cell lysates were subjected to immunoblot analysis for p-MET (Y1234/Y1235). **(B)** MDA-MB-231 cells treated with or without inhibitor at 10 µM were stimulated with HGF for 6h. Cells were subjected to immunofluorescence staining for ARHGEF7 and CDCP1. Scale bar, 10 µm and 2 µm (inset).

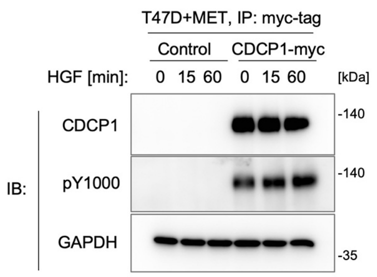

Fig. S6. Activation of CDCP1 by HGF stimulation.

CDCP1-myc was immunoprecipitated (IP) from T47D cells expressing MET with or without CDCP1-myc, and subjected to immunoblot analysis (IB) for CDCP1-myc and phosphorylated tyrosine (pY1000).

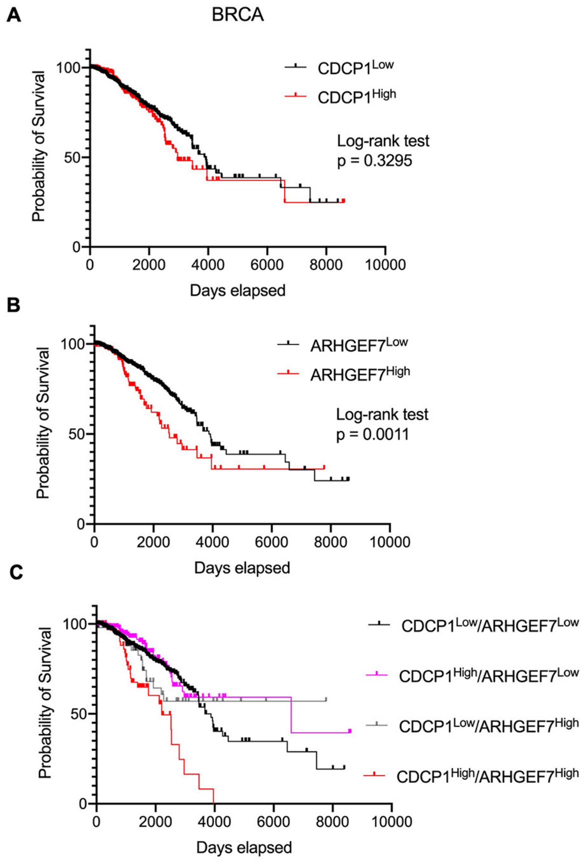

Fig.S7. Upregulation of ARHGEF7 correlates with poor prognosis in patients with breast cancer.

(**A-C**) mRNA expression of CDCP1 and ARHGEF7 was analyzed by using cohort of breast cancer patients (BRCA; n = 1209) from The Cancer Genome Atlas. Kaplan-Meier survival curves of breast cancer patients were constructed using mean expression (high vs low) and analyzed with the log-rank test. Patient number (Median survival days) are as follows: **(A)** CDCP1^{Low}: 893 (3926), CDCP1^{High}: 316 (2965), **(B)** ARHGEF7^{Low}: 1045 (3873), ARHGEF7^{High}: 164 (2534), CDCP1^{Low}/ARHGEF7^{Low}: 796 (3873), **(C)** CDCP1^{High}/ARHGEF7^{Low}: 249 (6593), CDCP1^{Low}/ARHGEF7^{High}: 97 (undefined), CDCP1^{High}/ARHGEF7^{High}: 67 (2207).
